# Effectiveness of cognitive behavioral therapy: An evaluation of therapies provided by trainees at a university psychotherapy training center

**DOI:** 10.1002/pchj.23

**Published:** 2013-05-30

**Authors:** Arto J Hiltunen, Elo Kocys, Renée Perrin-Wallqvist

**Affiliations:** Department of Psychology, Karlstad UniversityKarlstad, Sweden

**Keywords:** cognitive behavioral therapy, satisfaction with therapy, symptom relief, trainee therapists

## Abstract

At the psychotherapy training center at Karlstad University, a study was carried out to examine the levels of symptom change and satisfaction with therapy in a heterogeneous population of clients treated using cognitive behavioral therapy (CBT) by less experienced trainee therapists with limited theoretical education. The clients received an average of 11 therapy sessions. The results suggested that CBT performed by less experienced trainee therapists can be effective. According to client estimations, a statistically significant reduction in symptoms, measured using the Symptoms Checklist, was achieved for seven of nine variables (p ≤ .006), as well as a significant increase in satisfaction with life (p ≤ .001). Also, the pre- and posttherapy measurements using the Montgomery–Åsberg Depression Rating Scale showed a statistically significant improvement in the clients’ condition. According to the therapists’ estimations, 64% (SD = 32.01) of the clients experienced a significant improvement in their condition. In addition, the results of a survey of client satisfaction demonstrated that the clients were very pleased with the therapy received. Also the therapists were, to a great extent, satisfied with the treatment process itself, including the supervision received, and very satisfied with the client alliance. A correlation analysis between the clients’ perceived level of improvement and therapist satisfaction showed a strong correlation between the two variables (r = .50, p < .005). By including the Comparative Psychotherapy Process Scale (CPPS) in our study it was possible to measure trueness to therapy form. An analysis of the CPPS results confirmed that the form of therapy used at the training site was more strongly CBT than psychodynamic interpersonal treatment (p ≤ .001). The CBT subscale score indicated that the therapy was characteristic of CBT, confirming that the interventions used in the therapy belong to the CBT genre.

In today’s society, a significant gap exists between the demand for psychotherapy services and the available supply. One way to cope with this lack of delivery of psychological services could be step care models (Bower & Gilbody, 2005). Step care models are usually carried out for low-intensity treatment in the form of “minimal interventions” or brief therapies (Scott, Tacchi, Jones, & Scott, 1997), group treatments (Dowrick et al., 2000), or as self-help approaches such as bibliotherapy (Cuijpers, 1997) or computerized treatments (Proudfoot et al., 2004). One such potentially useful Step care model is the application of cognitive behavioral therapy (CBT) by trainee therapists.

Cognitive behavioral therapy, which has been defined by Roth and Fonagy (2005) as a “focus on how these maladaptive aspects of functioning are maintained by the individual’s environment and through properties inherent to his/her belief systems” (p. 8), is one of the most studied forms of psychotherapy, with empirical support for a number of psychological disorders. A meta-analysis, which included 16 studies, clearly showed that CBT is effective for treatment in many problem areas (Butler, Chapman, Forman, & Beck, 2006; see also Nathan & Gorman, 2007; Roth & Fonagy, 2005). Approximately 80 distinct and empirically supported CBT techniques have been identified by O’Donohue and Fisher (2008) and shown to be effective for various problem areas, including anxiety disorder, depression, skills acquisition, parent training, enuresis, development of assertive skills, pain management, stress management, classroom management, insomnia, social skills training, and problem solving skills (O’Donohue & Fisher, 2008).

Several controlled studies have shown significant improvements in treatment outcome for CBT regardless of whether it was conducted in municipal care, in outpatient care, in academic/psychiatric hospitals, in private practices/clinics, or at a psychotherapy training center at a university clinic (Bados, Balaguer, & Saldaña, 2007a; Foa et al., 2005; Fortune, Gracey, Burke, & Rawson, 2005; Gillespie, Duffy, Hackmann, & Clark, 2002; Kendall, 1998).

Only a few studies have been carried out that focus on psychotherapy training centers and their activities. There are, however, some studies on the effects of CBT that has been carried out by therapists with limited theoretical education. The results showed that treatment conducted by less experienced therapists can also achieve good results (e.g., Öst, Karlstedt, & Widén, 2012). In a study recently published by Öst et al. (2012), clients suffering primarily from anxiety disorders or depression improved significantly on both general dimensions as well as on disorder-specific measures. The outcome of the therapy provided by trainees was reported to be as good as the outcome achieved by experienced licensed cognitive behavioral therapists. Results from Lööf and Rosendahl (2010) indicate that the degree of symptoms for their clients was comparable with patients in general, and that both statistically and clinically significant improvement was achieved for the clients. Hence, these and other studies have demonstrated that CBT practiced by trainee therapists appears to be effective (Anderson & Lambert, 2001; Bados et al., 2007a; Foa et al., 2005; Lappalainen et al., 2007; Lööf & Rosendahl, 2010; Öst et al., 2012), even if the effect may be somewhat lower when compared with controlled studies.

In the studies in which therapist candidates have participated, the therapy sessions appear to be numerous. For example, the results of a Spanish study show that clients had 27.4 therapy sessions on average (Bados et al., 2007a). In contrast, no correlation between duration of therapy and recovery was found in a Swedish study of a psychotherapy training center (Lööf & Rosendahl, 2010). The dropout rate from therapy sessions may be somewhat higher when trainee therapists conduct the therapy (Bados et al., 2007a).

Several studies have focused on client satisfaction with CBT provided by trainees. The results have indicated that clients treated with CBT were satisfied with their treatment and would consider coming back to therapy again. In addition, the clients who achieved higher levels of symptom reduction rated their satisfaction with therapy more highly (Lööf & Rosendahl, 2010).

Everyday clinical praxis varies greatly, as psychotherapy is conducted by specialists with different educational levels and experiences. The various authorities that regulate Sweden’s health policy are, however, of the opinion that the availability of CBT ought to be increased. As pointed out by the Swedish National Board of Health and Welfare, as well as other parties involved, there is currently a great shortage of treatment personnel with adequate competency within this realm. To contribute to solving this problem, the Department of Psychology at Karlstad University initiated a master’s program in psychotherapy with a focus on CBT, for which the therapists are trainees and the psychotherapy module is part of their master’s degree. Trainees who are enrolled in the master’s program (Master in Case Management, 120 European Credit Transfer and Accumulation System [ECTS] credits) already hold a bachelor degree with a major in psychology and have studied CBT full-time for one term prior to the psychotherapy treatment sessions conducted during Terms 2–4 of the master’s program. The present work attempts to evaluate the low-intensity treatment carried out by trainee therapists with limited theoretical education and utilizing a CBT training model as a potential step care model for psychotherapy, which could serve as another utility with which to reduce the gap between the demand for psychological therapy services and the available supply.

The aim of this study was to examine the level of symptom change and satisfaction with therapy in a heterogeneous population of clients treated using CBT by less experienced trainee therapists with limited theoretical education. The specific goal was to investigate any possible changes regarding symptom level, as well as the level of satisfaction with the therapy they received. Also, therapist satisfaction with the treatment process and their supervision was to be measured.

## Method

This study was carried out within the framework of the Master in Case Management program with an emphasis on CBT at Karlstad University. It was a quantitative study with pre- and posttreatment assessments and a single-group design. Quality assurance is practiced at the Department of Psychology. Hence, the clients’ level of symptom change and satisfaction were studied in connection to the psychotherapy treatment. This study was conducted in the field and in accordance with the established criteria that apply to clinical practice.

### Participants

Both clients and therapists participated in the ratings of the therapy sessions and the outcomes. Clients were recruited through advertisements on the University website, which stated that free psychotherapy was offered at Karlstad University to clients with minor problems. This offer was primarily directed at students, but others seeking help were also treated if there were vacancies.

A total of 20 trainee therapists aged 24–64 years (*M* = 37.4 years, *SD* = 12.2 years, *Mdn* = 33.5 years) participated in the treatment. On average, each therapist treated two clients. The prerequisites for carrying out the therapy sessions were that the trainee therapist had a bachelor degree with a major in psychology, and had studied CBT for one term (15 ECTS credits in the form of lectures and workshops) as part of the Master in Case Management, obtaining a passing grade on the theoretical component. The program is mainly conducted via distance education, with on-campus meetings of 2–3 days duration run every third week. In all, there are 10–12 on-campus seminars per term (80–96 hr per term). The CBT education program consists of lectures, group projects, and examinations, in order to teach the trainees and evaluate their ability to diagnose the patients, to do a functional analysis, and to understand and carry out basic CBT techniques in their clinical practice (O’Donohue & Fisher, 2008).

The trainee therapists collected basic information about the clients. The client data comes from two different sources: from self-rating instruments filled out by the clients themselves and from therapist rating forms filled out by the therapists. Thus the data might differ somewhat. The self-rating instrument was filled out at the preassessment stage by 42 participating clients and at the postassessment stage by 35 (83.3%) clients. Regarding the therapists’ ratings, de-identified data were collected for 35 clients.

The demographic and clinical data listed in Table 1 come from the therapists’ rating forms. In all, 35 clients completed the treatment: 24 women and 11 men, with a mean age of 31.6 years. The clients had 11 therapy sessions on average. The therapy was based on a functional analysis set by the therapist in consultation with their supervisor. The clients were divided into six different diagnostic groups depending on their problem areas. Specific psychiatric diagnoses were not made because the possible treatment interventions were problem focused, not syndrome focused.

**Table 1:** Clients’ Demographic and Clinical Data

Variable	*N* = 35
Sex	
Female	24 (68.6%)
Male	11 (31.4%)
Age (years)	
Interval	19−78
*M* (*SD*)	31.6 (12.4)
*Mdn*	26
Problem areas	
Depression	1 (2.9%)
Anxiety	8 (22.9%)
Self-esteem	2 (5.7%)
Physiological problems	2 (5.7%)^a^
Multiple problems	18 (51.4%)^b^
Other	4 (11.4%)^c^
Number of therapy sessions	
Interval	4–31
*M* (*SD*)	11.2 (6.6)
*Mdn*	10

a Physiological problems refer to stress and migraine.

b Multiple problems can include some of the following areas: depression, anxiety, self-esteem, physiological problems, but also relationship problems, lack of ability, personality disorders, neuropsychiatric conditions, sleeping problems, and aggression.

c Other can include crisis, aggression, insomnia, lack of ability, and personality disorders.

### Design

The study began in January 2010 and the new clients started their therapy as vacancies occurred. Each therapist had one client at a time, who came from the psychotherapy training center’s own queue. The therapy was conducted individually and in the form of regular therapy sessions once per week for three terms at the most (Terms 2–4). Each session lasted for approximately 45 min. Participation was free and neither the clients nor the therapists were given any economic compensation.

The trainee therapists were supervised by qualified psychotherapists who were trained supervisors with many years of experience as CBT supervisors. The therapy sessions were recorded using audio or video. The supervisors could watch or listen to the recordings and, for educational purposes, other members of the same supervision group were allowed to watch or listen to each other’s recordings. Supervision in groups of four trainees was conducted once every third week for 40 hr per term. Each meeting lasted for 3.0–3.5 hr.

Most of the trainee therapists met their clients at the psychotherapy training center at Karlstad University. For practical reasons, however, therapy sessions were also conducted elsewhere in Sweden, depending on where the therapists lived, and then usually carried out in their workplace.

At the beginning of each client contact, the client, together with the therapist, signed a client information form stating the rules that would apply. The document contained information about the therapist, the therapy process, the number of sessions, recordings, confidentiality, supervision, and cancellations. Clients also gave their written consent to participate in the evaluation of the activities at the training site.

Characteristic of the entire treatment process was that the psychotherapy was not based on a manual. Rather, the trainees were provided with teaching, training, supervision of behavioral and functional analysis, and corresponding treatment methods, based on textbook descriptions of cognitive and behavioral interventions (O’Donohue & Fisher, 2008). Specific psychiatric diagnoses were not generated and the treatment was problem focused rather than syndrome focused.

### Instruments

Within the framework of this study the clients filled out pre- and posttreatment forms containing four rating scales: the Symptoms Checklist (SCL-90), the Montgomery–Åsberg Depression Scale (MADRS), the Satisfaction with Life Scale (SWLS) and the Rosenberg Self-Esteem Scale (RSE). When the therapy ended, the clients also filled out two scales measuring client satisfaction: the Client Satisfaction Questionnaire (CSQ-8) and a quality characteristics scale, the Comparative Psychotherapy Process Scale (CPPS). The therapists answered a therapist rating instrument. The rating scales were used as a complement to other diagnostic instruments and for evaluating treatment effectiveness.

The SCL-90 is a self-rating form with a multidimensional symptom profile and is a commonly used symptom rating scale (Derogatis & Cleary, 1977; Derogatis, Lipman, Rickels, Uhlenhuth, & Covi, 1974). A Swedish standardization has been done by Fridell, Cesarec, Johansson, and Malling Thorsen (2002). It is a self-rating instrument whereby the person rates their physical and mental health over the past week. The 90 questions of which the scale consists are divided into nine diagnostic and three general subscales. The nine subscales are: Somatization, Obsessive–Compulsive, Interpersonal Sensitivity, Depression, Anxiety, Hostility, Phobic Anxiety, Paranoid Ideation, and Psychoticism. The three global scales that measure superior aspects of general distress are the General Severity Index (GSI), the Positive Symptom Distress Index (PSDI), and the Positive Symptom Total (PST) (Derogatis et al., 1974; Derogatis & Cleary, 1977; Fridell et al., 2002).

In 1979, Montgomery and Åsberg (1979) introduced the rating instrument MADRS, which is based on ratings by British and Swedish patients. The purpose of the scale is to get a picture of the patient’s current state of mind by having them rate how they have felt during the past 3 days. The form contains nine statements about state of mind, anxiety, sleep, appetite, ability to concentrate, ability to take initiative, emotional commitment, pessimism, and zest for life (Montgomery & Åsberg, 1979).

The SWLS was developed by Diener, Emmons, Larsen, and Griffin (1985) in order to measure general satisfaction with life. The scale consists of five different statements such as: “In most ways my life is close to my ideal” and “The conditions of my life are excellent.” On a scale from 1 to 7, the respondent rates how well each statement corresponds to his or her life situation.

The RSE (Rosenberg, 1965, 1979) is one of the most frequently used self-rating instruments for evaluating a person’s self-esteem, and has high internal reliability. The instrument measures a positive or negative global self-concept. The scale includes 10 items and the respondent is asked to answer every statement and select the most suitable answer from 1 (*strongly agree*) to 4 (*strongly disagree*).

The CSQ-8 is used for measuring satisfaction with the therapy received as seen from the client’s perspective (Attkisson & Greenfield, 1999; Attkisson & Zwick, 1982). This self-rating instrument consists of eight items that all deal with health and human services, and can be used in a number of cases. Each item on the CSQ-8 is scored on a 4-point Likert scale, on which the answer alternatives vary greatly.

The CPPS was designed by Hilsenroth, Blagys, Ackerman, Bonge, and Blais (2005) to assess the distinctive features of psychodynamic interpersonal treatment (PDT) and cognitive behavioral treatments. The instrument includes two subscales: one measures PDT functions and the other measures CBT functions. Both subscales measure therapist activity and the techniques used during therapy sessions. The PDT subscale is used to measure those therapist activities and techniques that the PDT focuses on to a greater extent than does the CBT. In turn, the CBT subscale is used to measure those techniques and therapist activities that are stressed more in CBT than in PDT. The instruments consist of 20 randomly distributed items scored on a 7-point Likert scale ranging from 0 (*not at all characteristic*) to 6 (*extremely characteristic*). Hilsenroth et al. (2005) have demonstrated the ability of the scale to distinguish between PDT and CBT. The authors suggest that the CPPS has good inter-rater reliability, internal consistency, and promising validity. The scale and subscale items compare favorably to the reliability of other reported therapist activities. According to Hilsenroth et al.,

The psychometric features of the CPPS were consistent with the a priori, empirically based conceptualization of psychotherapy process designed to differentiate the session characteristics of PI and CB approaches to treatment. The CPPS identifies and operationally defines some of the theoretically central and distinctive activities of nonmanualized PI and CB therapy.(p. 350)

#### Rating instruments for the therapists

A simple questionnaire was created whereby the therapists answered questions about their clients. The questionnaire had items about the clients’ age, sex, problem picture, length of therapy, degree of client recovery, and therapy dropout, as well as any reasons for dropping out and at which phase the dropout occurred. Furthermore, the therapists evaluated to what extent they were satisfied with the treatment in general, the client alliance, and the supervision offered during the time of the treatment. The respondents rated their experiences on a 5-point Likert scale where 5 equaled *very satisfied* and 1 equaled *not satisfied at all*.

All instruments except the therapist’s rating instrument have been used in previous studies and have good psychometric properties (Diener et al., 1985; Hilsenroth et al., 2005; Larsen, Attkisson, Hargreaves, & Nguyen, 1979; Rosenberg, 1979; Silber & Tippett, 1965).

### Methods of collecting data

The data analyzed came from four different investigations of client symptom ratings, client satisfaction, quality characteristics measures, and therapist evaluations. A standard battery of the following self-rating scales was filled out by the clients: the SCL-90, the MADRS, the SWLS, and the RSE. The pretest measurements were carried out during the admission session and the posttest measurements were conducted after the last treatment session. The CSQ-8 and the CPPS were handed out at the end of the course of therapy and the clients filled them out anonymously. The therapists, in turn, filled out a therapist rating instrument specifically designed for this study. The questionnaires were distributed on completion of the therapy and then collected for further analysis.

### Procedure

Completed forms for the SCL-90, the MADRS, the SWLS, and the RSE were compiled by the trainee therapists, signed by the supervisor, and handed to the center for future evaluation of the activities.

The clients themselves sent the completed questionnaires regarding client satisfaction (the CSQ-8) and the quality characteristics measure (the CPPS) by mail, in prestamped envelopes, to the secretary of the psychotherapy training center. The trainee therapists did not have access to the clients’ answers, which were then de-identified so that all information which could have identified the clients was removed. Clients had to give their written consent so that the Department of Psychology could use these questionnaires to evaluate the activities.

Participation in the therapist’s survey was voluntary. The therapists handed in or sent the completed forms to the research leader.

### Statistics

The processing of data was conducted with the aid of the statistics program, SPSS. A one-way repeated-measures ANOVA *F*-test was used to analyze symptom reduction. Here, the means of the pre- and posttest scores for the MADRS, the SWLS, the RSE, and the SCL-90 were compared, including all subscales. Only data from the first client for each therapist were used in the analysis. The Blom transformation was used, as the data were not normally distributed (Blom, 1958), and the Bonferroni correction was applied to prevent a mass significance problem.

To explore to what extent the clients were satisfied with the therapy, the mean value of client satisfaction with therapy (the CSQ-8) was calculated and compared with the equivalent results from other studies.

A dependent *t*-test analysis of the quality characteristics scale (the CPPS) was conducted to confirm that the therapy performed was CBT.

The mean value of the therapists’ satisfaction with treatment, the client alliance, and their supervision was calculated in order to examine whether or not the therapists were satisfied with the treatment, the client alliance, and the supervision that was provided for them during the therapy sessions.

Finally, a correlation analysis was carried out to see if a correlation existed between the clients’ level of improvement and the number of therapy sessions in which they had participated. For this purpose, data were used from the therapist rating scales whereby the therapists evaluated their perceived experience of to what extent their clients’ condition had improved.

## Results

### Dropouts

Rating results from 35 clients were obtained through the therapist’s questionnaire. The results showed that five persons (14.3%) dropped out of therapy prior to the end of the treatment. The reasons stated for dropping out were: busyness, lack of motivation, problems with their own children, moving abroad, and considered oneself to have recovered. Three of the clients dropped out of therapy in the preparatory phase, one in the treatment phase and one in the completion phase.

### Comparative analysis of the participants’ symptom level before and after therapy

**Figure 1:**
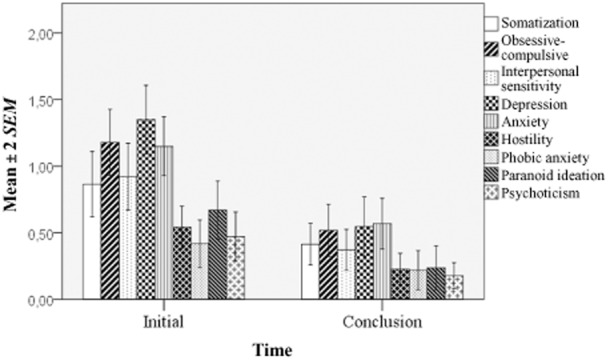
The clients’ mean score for the Symptoms Checklist (SCL-90) subscales before and after therapy.

**Figure 2:**
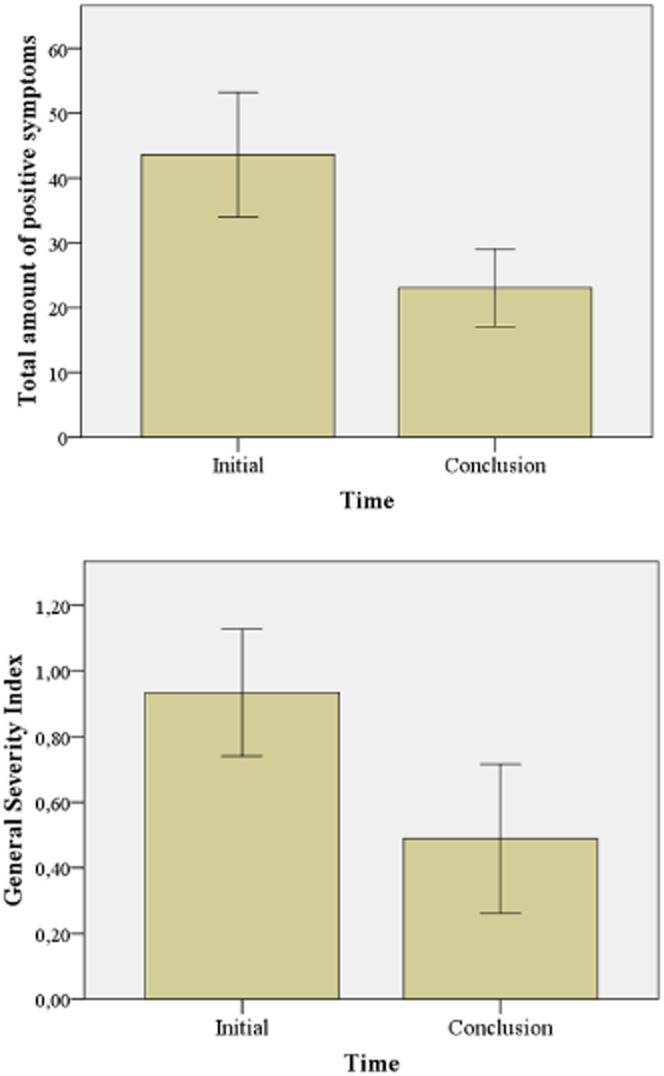
The clients’ mean score for the Symptoms Checklist (SCL-90) global subscales, the General Severity Index (GSI), and the Total amount of Positive Symptoms (PST) before and after therapy.

In most cases, the results of the participants for all subscales on the SCL-90 showed a great improvement from the time when the client started the therapy until it was completed (Figure 1). Generally, the clients’ level of mental distress had been significantly reduced according to the before-and-after measurements. A one-way ANOVA showed a statistically significant level of symptom reduction for a majority of the mean values on the SCL-90 subscales. The effects were significant for seven of nine subscales as follows: Somatization, *F*(1, 13) = 9.7, *p* < .01; Obsessive–Compulsive, *F*(1, 13) = 19.2, *p* < .001; Interpersonal Sensitivity, *F*(1, 13) = 3.8, *p* < .01; Depression, *F*(1, 13) = 19.3, *p* < .001; Anxiety, *F*(1, 13) = 10.6, *p* < .01; Hostility, *F*(1, 13) = 4.9, *p* < .05; and Paranoid Ideation, *F*(1, 13) = 7.1, *p* < .05. No statistically significant difference was found for the results of the symptom ratings before and after therapy for the SCL-90 subscales of Phobic Anxiety, *F*(1, 13) = 1.5, *ns*, and Psychoticism, *F*(1, 13) = 1.9, *ns*. Further, no statistically significant difference was found for either the PSDI, *F*(1, 13) *=* 3.5, *ns*, or the GSI, *F*(1, 13) = 4.1, *ns*. However, one of the three global subscales, the PST, showed a statistically significant symptom reduction, *F*(1, 13) = 15.5, *p* < .005 (Figure 2).

**Figure 3:**
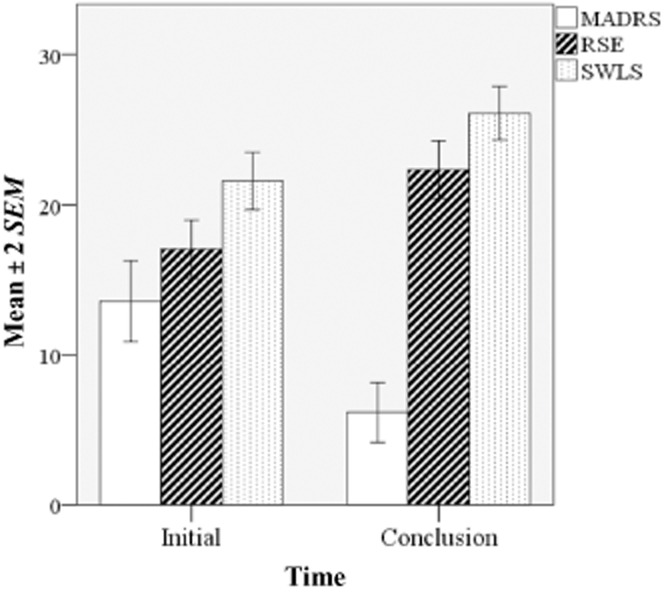
The clients’ mean scores for the Montgomery–Åsberg Depression Scale (MADRS), the Rosenberg Self-Esteem Scale (RSE), and the Satisfaction with Life Scale (SWLS) before and after therapy.

Further, the other measurement instruments showed significant effects with regard to the pre- and posttreatment measurements. The symptom levels experienced by the clients had decreased significantly for the MADRS, *F*(1, 13) = 18.3, *p* < .001, and the SWLS, *F*(1, 13) = 17.3, *p* < .005. No significant improvements regarding self-esteem were found using the RSE, *F*(1, 13) = 3.8, *ns.* The results illustrate that the level off depression experienced by the clients decreased drastically and their satisfaction with life improved markedly (Figure 3).

### Client satisfaction with therapy

In all, 25 participants answered the CSQ-8 questionnaire regarding satisfaction with the therapy. The analysis of the results for the CSQ-8 showed that the clients’ mean rating of satisfaction was 29.1 (*SD* = 2.7, *Mdn* = 30.0, range = 8–32). The results showed that the clients were very satisfied with the therapy.

### Therapist’s ratings of clients’ improvement level

The therapist’s ratings of the extent to which their clients got better confirmed the above results. According to the data reported by the therapists, their clients became 64% (*SD* = 32.0%) better after an average of 11.2 (*SD* = 6.6) therapy sessions. Further analysis showed that the condition of 70% of the participants improved by 65.6% on average, that is, more than two-thirds of the clients reached a very good result. The most frequently reported level of improvement was 90%, which was reported ten times in total (28.6%).

### Therapist’s satisfaction with therapy, client alliance, and supervision

In this study, the therapists answered questions about their satisfaction with the treatment in general, as well as with their supervision, and the perceived therapist–client alliance. The results showed that, on average, the participants had fairly high ratings of their satisfaction with the treatment, the client alliance, and their supervision. These results imply that the therapists were satisfied with the treatment process and the supervision generally, and were very satisfied with the therapist–client alliance (Table 2).

**Table 2:** Therapist Satisfaction with Treatment, Alliance, and Supervision

*N* = 34	*M*	*SD*	*Mdn*	Minimum	Maximum
Treatment	3.97	1.14	4.0	2.0	5.0
Alliance	4.26	0.79	4.0	2.0	5.0
Supervision	3.94	0.85	4.0	1.0	5.0

*Note*. Scale interval = 1–5.

### Correlation between level of improvement and satisfaction with supervision

A correlation analysis between the perceived level of improvement and the level of therapist satisfaction demonstrated a strong correlation between the two variables (*r* = .50, *p* < .005). This means that, for the therapists, there was a strong positive correlation between the perceived level of client improvement and how satisfied the therapists themselves were with the supervision. The therapists who rated their clients’ level of improvement more highly were also more satisfied with their supervision.

### Correlation between level of improvement and number of sessions

A correlation analysis between the client’s level of improvement and the number of therapy sessions showed no correlation between the two variables (*r* = .08, *ns*). A possible interpretation could be that the number of therapy sessions did not affect the client’s improvement level, but in order to draw such conclusions an experimental study, which was not part of this project, needs to be conducted.

### Comparative forms of psychotherapy

A paired samples *t*-test showed that the difference between the CBT and PDT variables was significant, *t*(22) = 4.0, *p* ≤ .001. These results implied that the form of therapy received was CBT (Table 3).

**Table 3:** Comparative Analysis of Received Form of Therapy

*n* = 23^a^	*M*	*SD*	*Mdn*	Minimum	Maximum
Cognitive behavioral therapy	4.1	0.92	4.3	2.3	5.9
Psychodynamic interpersonal treatment	3.5	0.93	3.4	1.3	5.1

a The difference in the number of participants is due to two clients not filling out the Comparative Psychotherapy Process Scale form. Hence, the analysis includes data from 23 participants.

## Discussion

The purpose of this study was threefold: to evaluate the trainee therapists’ treatment interventions, to study possible changes in the level of symptoms, and to evaluate the satisfaction with therapy of a heterogeneous group of clients who were treated using CBT at a training site. Furthermore, this study was also designed to evaluate client and therapist satisfaction, and to make a contribution towards a step care model to reduce the gap between the demand for psychotherapy services and the available supply.

The current study shows that the trainee therapists who had participated in supervision and in the theoretical course on CBT methods were able to produce a good treatment result for their clients, within relatively short treatment times. The analysis indicates that the clients who completed the therapy gained a significant symptom reduction and their quality of life improved considerably. One interpretation of the statistically significant results that were obtained for seven of nine SCL-90 subscales, as well as for one global scale at pre- and posttreatment measurements, is that the clients experienced a considerable improvement. A possible explanation for why the subscales of Phobic Anxiety and Psychoticism did not show any statistically significant results for symptom relief could be that the participants gave the lowest scores on these scales at the beginning of the treatment. Hence there was a limited possibility of reducing these symptoms even further.

The pre- and posttherapy measurements of the MADRS and the SWLS also showed a statistically significant improvement in the clients’ condition, as well as improved quality of life. This data is in line with previous research results that have shown CBT to be an effective form of treatment for clients suffering from moderate mental distress (Butler et al., 2006; Fortune et al., 2005; García-Palacios et al., 2002; Gillespie et al., 2002; Nathan & Gorman, 2007; Roth & Fonagy, 2005; Westbrook & Kirk, 2005). Further, the described treatment carried out at the University’s psychotherapy training center seemed to be effective regarding client recovery. These results are comparable with some other studies in which trainees have practiced CBT with significant results (Anderson & Lambert, 2001; Bados et al., 2007a; Foa et al., 2005; Lappalainen et al., 2007; Lööf & Rosendahl, 2010; Öst et al., 2012). Several authors have stressed the high efficacy found in controlled randomized studies, as well as in studies conducted by experienced therapists. For this reason, the current study, along with that of Öst et al., provides evidence that therapists without much experience in clinical practice can achieve results as good as those produced by experienced therapists.

Perhaps one could argue that, because the participants were a highly heterogeneous group with different “minor problems,” it is hard to see how the outcome measures could pick up the intended or expected change. The Swedish version of the SCL-90 has been validated against several patient populations and shows good discriminant and convergent validity in the classification of control and patient populations (Fridell et al., 2002). Further, when four diagnostic groups are compared (anxiety disorder, atypical psychosis, affective disorder, and not-otherwise-specified depression/neuroticism), anxiety disorder has higher scores on the SCL-90 compared with the three other diagnoses; the same is true also for the index scales. Therefore, we believe that the SCL-90 and the complementary scales (the SWLS, the RSE, and the MADRS) used in our study had very good correspondence to the primary problem areas seen in our clients. While we recognize the need to carry out analysis at levels more specific than that of a heterogeneous group, we believe the sample size in the current study is too limited to permit such an attempt here.

The question as to why the treatment provided by trainee therapists, as shown in the present study, as well as by Öst et al. (2012), seems as effective as the treatment provided by experienced therapists is something that should be discussed. The therapists that participated in this study had different backgrounds with regard to education and work experience. All the therapists had studied the theory of CBT for one term prior to working with the clients and, along with the clinical work with the clients, another three terms of theoretical studies were offered. All trainee therapists had a bachelor degree with a major in psychology, because this was a prerequisite for admission to the master’s program. After an average of 11 therapy sessions, 64% of the clients improved their mental health. This result is along the lines of previous research results, such as those of Hansen, Lambert, and Forman (2002) who found that the mean number of treatment sessions provided for clients in controlled randomized studies was 12.7, and 58% of the clients obtained a clinically significant result. Anderson and Lambert (2001) suggest that it takes somewhere between 11 and 16 sessions for 50% of the clients to achieve a clinically significant change. That is slightly fewer sessions compared with other suggestions for CBT, which recommend 15–20 sessions (Kåver, 2006). At the same time, however, some trainee research shows that psychotherapy performed by trainee therapists tends to last somewhat longer, as also concluded in a study by Bados et al. (2007a). In that study, the mean number of therapy sessions was 27, but the proportions of clients who improved their health and recovered were as high as in other studies, 61% and 52%, respectively. The study by Lööf and Rosendahl (2010) at a training center in Lund demonstrated that 67% of the clients experienced a reliable improvement of their mental health and 28% obtained a clinically significant improvement after an average of 6 months of therapy, which is the equivalent of approximately 20 therapy sessions. To conclude, the results of our study thus seem to correspond more closely with studies in which more experienced therapists participated than with those in which trainee therapists took part.

The correlation analysis performed in order to examine the perceived correlation between the clients’ level of improvement and the therapists’ satisfaction with their supervision showed a strong correlation between the two variables: the therapists who rated their client’s level of improvement higher were more satisfied with their supervision. One explanation as to why the therapy performed by inexperienced therapists could get such high results for symptom reduction may be the availability of supervision, and the high quality of the supervision could be a contributing cause as to why the clients obtained a high level of improvement. Also Roth and Fonagy (2005) stress the impact that supervision has on the efficacy of psychotherapy.

Orlinsky, Grawe, and Parks (1994) found a positive correlation between the length of treatment and the results in 64% of the studies that they examined. However, no statistically significant correlation was found between the clients’ level of improvement and the number of therapy sessions, neither in our study nor in some previous studies (Hansen et al., 2002; Lööf & Rosendahl, 2010). It should be considered that the length of treatment depends on the client’s issues and problems. CBT is considered particularly effective for mild and moderate mental distress. Consequently, clients with mental disorders or more complicated problems might influence both the length of the therapy and the treatment outcome. The present results suggest that a significant symptom reduction can be achieved after approximately 11 sessions. The design of this study does not, however, provide us with the possibility to draw conclusions regarding the length of treatment and the treatment result, that is, whether or not the clients who received more sessions experienced a greater symptom reduction, as the participants represented a heterogeneous group of clients who differed from each other concerning both problems/issues and demographic data, and no formal diagnoses were made. Treatment was based on the client’s request and the treatment structure, as well as on the functional and behavioral analyses conducted. Although the intention was to complete the treatment in one term, the number of therapy sessions depended on the needs of each client which, in turn, differed considerably. The length of treatment varied between four and 31 therapy sessions depending on the problem or problems. Many of the clients in this study had multiple problems that took slightly longer to treat. The clinic was a training center for therapists and this is probably a contributing cause as to why the number of therapy sessions varied. Although no conclusions can be drawn regarding cost-effectiveness, it can be stated that approximately one-third of the clients improved their mental health considerably after only approximately 11 therapy sessions.

There was not a high dropout rate for this study. On the contrary, a total of five persons (14%) dropped out of therapy, four in either the introductory phase or treatment phase, and only one in the termination phase. Hence our result does not correspond with other studies, for which the therapy dropout rate tends to be much higher. Bados, Balaguer, and Saldaña (2007b) reported a 44% dropout rate for trainee therapist sessions and also noted that all clients in the study more often suffered from emotional disorders, eating disorders, or impulse control issues. In another study by Bados et al. (2007a), the dropout rate was slightly lower (33%), but still considerably higher than for the present study. High dropout rates could depend on the study design, that is, how the term *dropout* is defined. In the present study, the number of dropouts equaled the number of participants who terminated the treatment after having started the therapy. Those clients who had handed in an interest form and then withdrew it before the first session were not seen as dropouts. In the current study, four of the dropouts had multiple problems, one of whom quit therapy in the initial phase, and three who quit therapy in the treatment phase. The client who dropped out of therapy in the termination phase suffered from anxiety problems and the withdrawal coincided with the client moving abroad. We cannot draw any conclusions regarding the underlying causes for dropping out of therapy, but it is possible that mental codisorders make it more difficult to go through with the treatment. It probably did not suit all clients to undergo treatment due to their problems/issues.

The client satisfaction ratings (the CSQ-8) showed that the clients who underwent treatment rated high for satisfaction. These ratings were slightly higher than for several other studies. Lööf and Rosendahl (2010) reported approximately the same results in their study, which found that clients were satisfied with CBT (*M* = 27.7, *SD* = 3.6) and would consider going back to therapy again. Furthermore, the clients who achieved higher symptom reduction rated higher for satisfaction with therapy (Lööf & Rosendahl, 2010). Wise (2003) also noted greater improvements when participants indicated satisfaction with therapy (*M* = 28.8) and with clients who improved their mental health (*M* = 30.1). Hence, those results correspond with the results of the present study. According to Attkisson and Greenfield (1999), the results of the CSQ-8 are usually distributed unevenly, which mirrors the tendency of participants to report high levels of satisfaction, and the mean is frequently approximately 27, with standard deviations of approximately four. The CSQ-8 scores were slightly higher for this study compared with other studies, and also more homogeneous, that is, less spread out. The rating scale was filled out by 25 of the 35 persons who completed the treatment. According to the literature, the response frequency is seldom higher than 40–50% (Attkisson & Greenfield, 1999). When seen from this perspective, our study scores high above the mean value with a response frequency of 71%.

From this study, the conclusion can also be drawn that in general, the treatment performed at the university training center worked very well. The trainee therapists scored high on perceived satisfaction (Table 2). These scores indicate that the therapists themselves were relatively satisfied with the treatment process and their supervision, and were very satisfied with the client alliance. Even if the abovementioned analyses generally displayed very good results, none of them can answer the question as to whether or not the therapy form performed was indeed CBT. By including the CPPS rating scale in our study it was possible to measure the trueness to therapy form. The analysis demonstrated that there were significant differences between the CBT and the PDT (Table 3). The CPPS result indicated good quality characteristics and hence confirms that the interventions used in the therapy belonged to the CBT genre (Hilsenroth et al., 2005).

This study was carried out as a degree project within the framework of an evaluation and quality assurance project at Karlstad University, and therefore holds certain limitations due to the design used by the aforementioned research project. One key limitation is the lack of a control group, which makes it impossible to compare to what extent the clients’ level of improvement could be linked to the effectiveness of CBT versus to what extent spontaneous improvement occurred. Also, the study does not say anything about the durability of the effects. Therefore, further research on the long-term effects of CBT would be useful.

To conclude, the results of this study demonstrated that trainee therapists with training in CBT from the Karlstad University training center could provide significant symptom reduction and good treatment effectiveness.
